# Small molecules targeting LapB protein prevent *Listeria* attachment to catfish muscle

**DOI:** 10.1371/journal.pone.0189809

**Published:** 2017-12-18

**Authors:** Ali Akgul, Nawar Al-Janabi, Bhaskar Das, Mark Lawrence, Attila Karsi

**Affiliations:** 1 Department of Basic Sciences, College of Veterinary Medicine, Mississippi State University, Mississippi State, Mississippi, United States of America; 2 Departments of Medicine and Pharmacological Sciences, Icahn School of Medicine at Mount Sinai, New York, New York, United States of America; University of Campinas, BRAZIL

## Abstract

*Listeria monocytogenes* is a Gram-positive foodborne pathogen and the causative agent of listeriosis. *L*. *monocytogenes lapB* gene encodes a cell wall surface anchor protein, and mutation of this gene causes *Listeria* attenuation in mice. In this work, the potential role of *Listeria* LapB protein in catfish fillet attachment was investigated. To achieve this, boron-based small molecules designed to interfere with the active site of the *L*. *monocytogenes* LapB protein were developed, and their ability to prevent *L*. *monocytogenes* attachment to fish fillet was tested. Results indicated that seven out of nine different small molecules were effective in reducing the *Listeria* attachment to catfish fillets. Of these, three small molecules (SM3, SM5, and SM7) were highly effective in blocking *Listeria* attachment to catfish fillets. This study suggests an alternative strategy for reduction of *L*. *monocytogenes* contamination in fresh and frozen fish products.

## Introduction

*Listeria monocytogenes* is a Gram-positive foodborne pathogen causing listeriosis, which has a high mortality rate [1]. *L*. *monocytogenes* was discovered in the 1930s as a pathogen of animals and humans. It was initially identified as the cause of miscarriage in early pregnancy, stillbirth, and septicemia after an uneventful birth. *L*. *monocytogenes* occurs naturally in a wide variety of domestic animals, and it has been isolated from raw staple foods such as chicken, seafood, meat, and milk. *L*. *monocytogenes* is found on the external surfaces of fresh and frozen fish, as well as in the processing plant environment. *L*. *monocytogenes* shows elevated heat resistance, growth at refrigeration temperatures, tolerance to reduced pH, and growth in the presence of over 5% sodium chloride; therefore, it is a substantial foodborne pathogen in ready-to-eat aquaculture products [2–4]. Numerous studies have shown that over a quarter of frozen seafood was contaminated with *L*. *monocytogenes* [5, 6]. *L*. *monocytogenes* strains vary in their pathogenic potential [7–10]. Serotypes 4b, 1/2a, 1/2b, and 1/2c are considered highest risk, while serotypes 3a, 3b, 3c, 4a, 4c, 4d, and 4e are considered low-risk for listeriosis [11].

Cell surface anchor proteins are important in the attachment process of several bacterial species on food surfaces [12, 13]. These proteins are involved in bacterial adherence [14], and *L*. *monocytogenes* encodes a larger number of predicted surface proteins compared to other bacteria. It has 133 total predicted surface proteins that constitute 4.7% of its genome [15]. Examples include internalin A and B (InlA+InlB), which facilitate *Listeria* adhesion and invasion to mammalian cells [16, 17]. These proteins have LPXTG motif and Leucine-Rich Repeats (LRR) domains, which are used as cell wall anchors. Another cell wall protein, actin-binding protein (ActA), stimulates accumulation and polymerization of actin and helps in movement of *L*. *monocytogenes* from cell to cell during infection [18]. Additionally, cell wall-anchored peptidoglycan hydrolase (autolysin) play a role in *Listeria* virulence [19].

Boron-containing pharmacophore groups interact with a target protein not only through hydrogen bonds but also through irreversible covalent bonds, producing potent biological activity (i.e. antifungal, antiparasitic, protease inhibitors, etc.) [20]. They can be used as preventive, diagnostic, and therapeutic tools [21]. Boron-based compounds have the ability to reach many frequently targeted biomolecules in medicine, and boron is not considered toxic [21]. Examples of applications where they are used include cancer treatment [Velcade^®^ (bortezomib; Millenium Pharmaceuticals), which is FDA approved] and anti-fungal therapy [Kerydin^™^ (tavaborole; Anacor), which was approved by FDA in 2014].

LapB (Lmof2365_2117), a putative cell wall surface protein in *L*. *monocytogenes* strain F2365, has an orthologous protein from serovar 1/2a strain EGD-e (Lmo2085) that is significantly up-regulated in a murine macrophage cell line [22]. A nonvirulent catfish isolate (serotype 4a isolate HCC23, GenBank # NC_011660) [23] also encodes an orthologous protein (LmHCC_0465), but an ortholog is not encoded in the genome of *Listeria innocua* strain 11262. In our previous study, attenuation of *L*. *monocytogenes* strain F2365 LapB mutant (LmF2365Δ*2117*) was shown in mice. This mutant exhibited impaired adherence and replication intracellularly as well as reduced attachment capabilities to catfish fillet [24]. In the current study, the effects of boron-based small molecules designed to target the predicted active site of LapB protein were investigated. This strategy may provide an alternative method to reduce *L*. *monocytogenes* contamination in fresh and frozen fish products.

## Materials and methods

### Bacterial strains and growth conditions

*Listeria monocytogenes* strain F2365 (wild-type) and strain LmF2365Δ*2117* (LapB mutant) were cultured in brain heart infusion (BHI) agar or broth (Difco, Sparks, MD) and incubated at 30°C throughout the study.

### Preparation of catfish fillets

All fish experiments were conducted under a protocol approved by the Institutional Animal Care and Use Committee (IACUC) at Mississippi State University. Fillet attachment model was optimized using *Salmonella* chicken skin attachment model [25–27]. Briefly, specific-pathogen-free (SPF) channel catfish fingerlings were obtained from the College’s SPF fish hatchery and stocked in a 40-L tank with a continuous water flow and aeration. Water temperature was kept at 28 ± 2°C, and catfish were fed twice a day. Chlorine, dissolved oxygen, and temperature of the tanks were monitored daily. After one week of acclimation, catfish were euthanized in high dose MS-222 (400 mg/ml) (Argent Chemical Labs, Redmond, WA, USA), and a 6 mm biopsy punch was used to cut out uniform muscle samples.

### *Listeria monocytogenes* muscle attachment model

Six mm uniform muscle samples (3 to 5) were placed in 1.5 ml sterile centrifuge tubes. *L*. *monocytogenes* were grown to mid-log phase (OD_600_ 0.6–0.8) and diluted 10,000 times in phosphate buffered saline (PBS). Bacterial concentrations were determined by serial dilution and plate counting. 500 μl of diluted *L*. *monocytogenes* (~1x10^3^ CFU) were added to each muscle sample. The attachment was conducted at 30°C for 30 min, and unattached bacteria were removed by washing samples two times with 1 ml room temperature PBS by inverting tubes up and down ten times. A third wash was conducted on a shaker for 30 min at room temperature. After washing, muscle samples were homogenized in 250 μl PBS by a hand-held tissue homogenizer, and 750 μl PBS was added to the homogenate. Bacteria numbers were determined by serial dilution and plate counting. Experiments included ten replicates, and each experiment was repeated four times.

### Design of boron-based small molecules

Boron-based small molecules against *Listeria* LapB protein were developed by following previously published procedures [28–33]. Briefly, the amino acid sequences of *Listeria* LapB protein were searched against the European Bioinformatics Institute (EBI, http://www.ebi.ac.uk/) database for any existing homologous proteins, which found 147 hits. Filtering of the hits resulted in 26 hits, and finally, three collagen-binding surface proteins. Because these three proteins did not have any known inhibitors, in-house homology modeling tools, such as MolSoft ICM (Molsoft, San Diego, CA) and MOE (www.chemcomp.com), to determine the potential active site of the *Listeria* LapB protein. After this, the compounds were synthesized using the known reactions. Computational protein structure modeling programs (e.g., M4T, MMM, and Mutate) as well as standard programs (e.g., Autodock4, Surflex-Dock, ICM, PESD, SFC, SYBYL, etc.) were used to study the active site, and small molecules were fitted into this active site. The new compounds were characterized using proton (^1^H), carbon (^13^C) and high-resolution mass spectroscopy (HRMS), and the purity of the compounds was determined by high-performance liquid chromatography (HPLC). Synthesized small molecules were lyophilized in dark centrifuge tubes, wrapped in aluminum foil, and kept at room temperature until use. The stock solutions were prepared by dissolving small molecules in 1 ml DSMO, which were kept at 4°C.

### Small molecule effect on *Listeria monocytogenes* growth and muscle attachment

To test potential adverse effects of small molecules on *L*. *monocytogenes* growth and to determine the optimum concentration to be used in attachment experiments, 0, 5, 10, 25, and 50 μM solutions of all small molecules in BHI were prepared. At each concentration of all small molecules, four culture tubes were inoculated with *Listeria* (9 small molecules x 5 concentrations x 4 replicates = 180 cultures) and cultures were grown overnight at 30°C. OD_600_ values were measured, and average values at each concentration were calculated. Colony numbers were calculated at each concentration by serial dilution of cultures from randomly chosen four small molecules. After determination of the dose that is not affecting the growth of *L*. *monocytogenes*, all small molecules were tested following the *Listeria* muscle attachment model described above. The two experimental groups were muscle + *L*. *monocytogenes* + small molecules (treatment) and muscle + *L*. *monocytogenes* (control). Experiments were repeated four times, and each experiment included ten replicates.

### Statistical analysis

Normality of bacterial counts was checked by visual assessment of histograms using PROC UNIVARIATE in SAS for Windows 9.3 (SAS Institute, Inc., Cary, NC). When colony counts were not normally distributed, the log_10_ transformation was applied, and transformed data were analyzed by Student’s t test (P < 0.05).

## Results

### Boron-based small molecules

**Fig 1:**
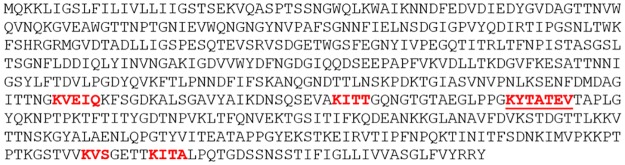
Potential active sites in the *Listeria monocytogenes* LapB protein. Potential active sites were shown as red and bold letters. Underlined letters indicate the most probable active site.

Homology modeling indicated the potential active sites of the *L*. *monocytogenes* LapB protein. In particular, amino acids sequences from 435 to 441 (KYTATEV) seem to be the most probable active site (Fig 1).

**Fig 2:**
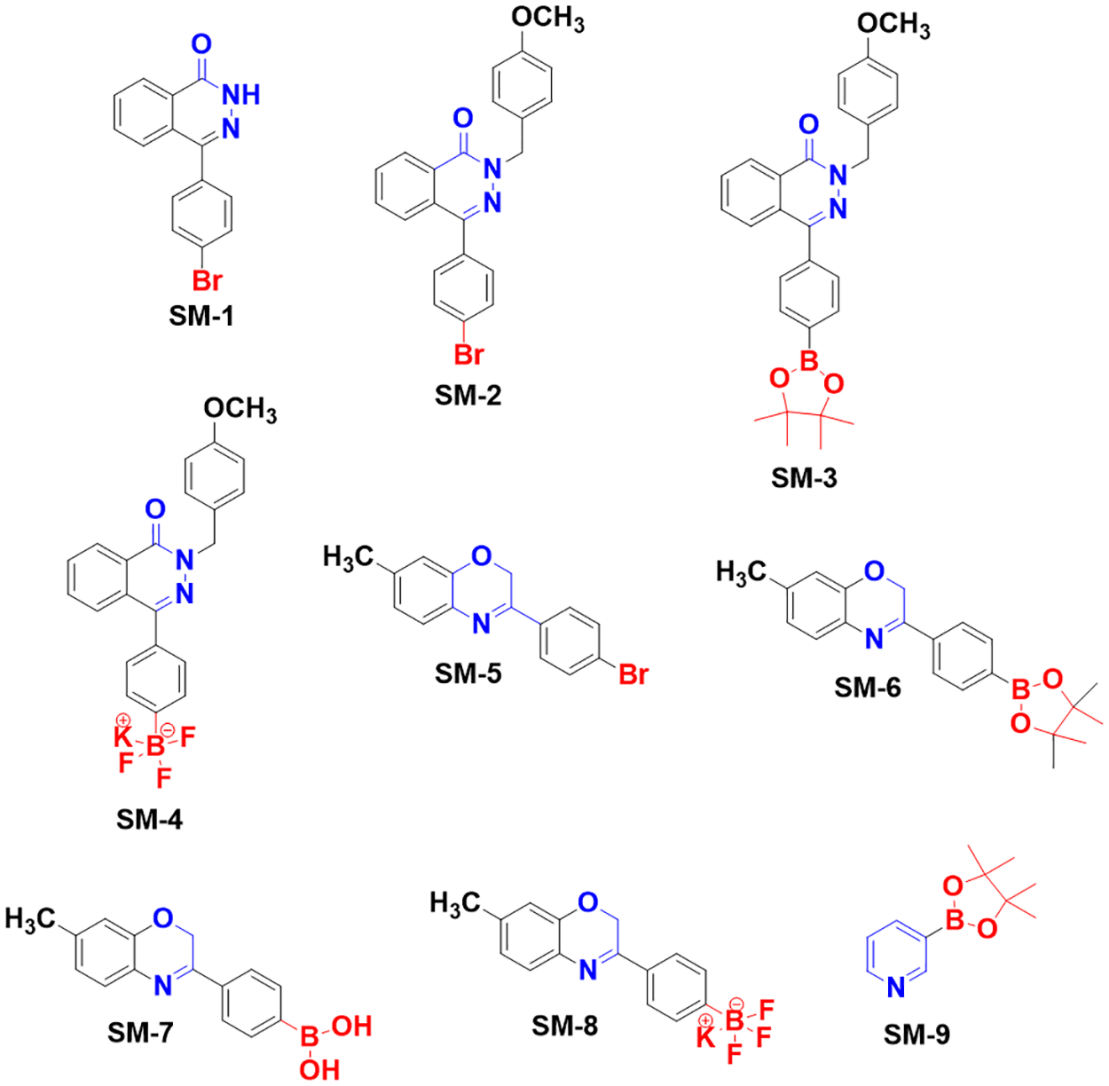
Boron-based small molecules designed against the *Listeria monocytogenes* LapB protein.

The small molecules (SM) were synthesized with different pharmacophore groups. SM1-4 are 2, 4-disubstituted-phthalazin-1(2H)-one derivatives with boron and without boronic acid potassium salts of trifluoroborate. SM5-8 are 3, 7 disubstituted-2H-benzo[b] [1, 4] oxazine derivatives, and SM6-9 are pyridine substituted boronic acid derivatives that mimic dipeptides. All compounds were above 98% pure, and exact structures are shown in Fig 2.

### Effect of boron-based small molecules on *Listeria monocytogenes* growth

**Fig 3:**
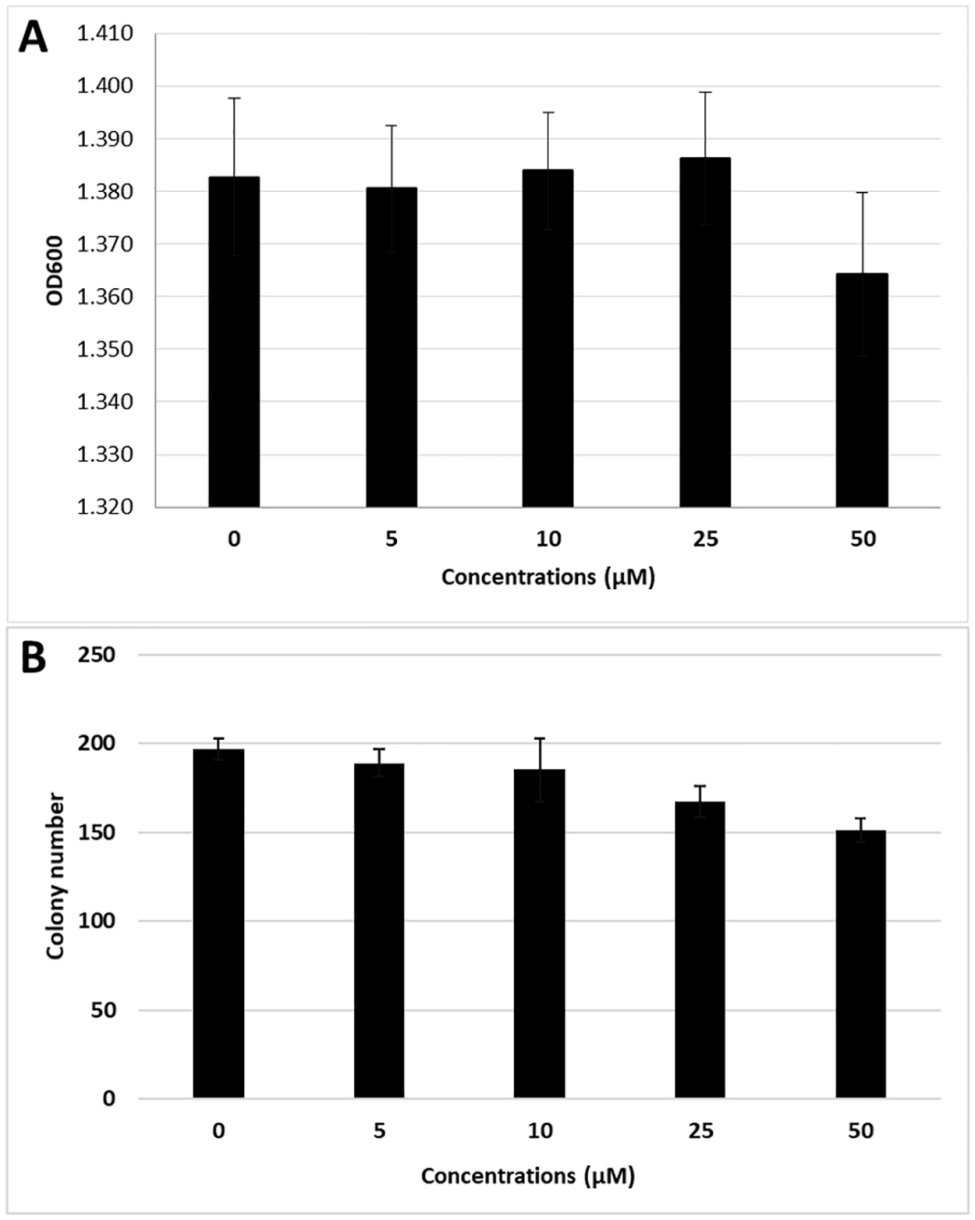
Effect of different concentrations of boron-based small molecules on *Listeria monocytogenes* growth. Growth comparison was conducted by OD measurement (A) and colony counts (B). At each concentration of all small molecules, four culture tubes were inoculated with *Listeria* (9 small molecules x 5 concentrations x 4 replicates = 180 cultures) and cultures were grown overnight at 30°C. OD_600_ values were measured, and average values at each concentration were calculated. Colony numbers were calculated at each concentration by serial dilution of cultures from randomly chosen four small molecules.

**Fig 4:**
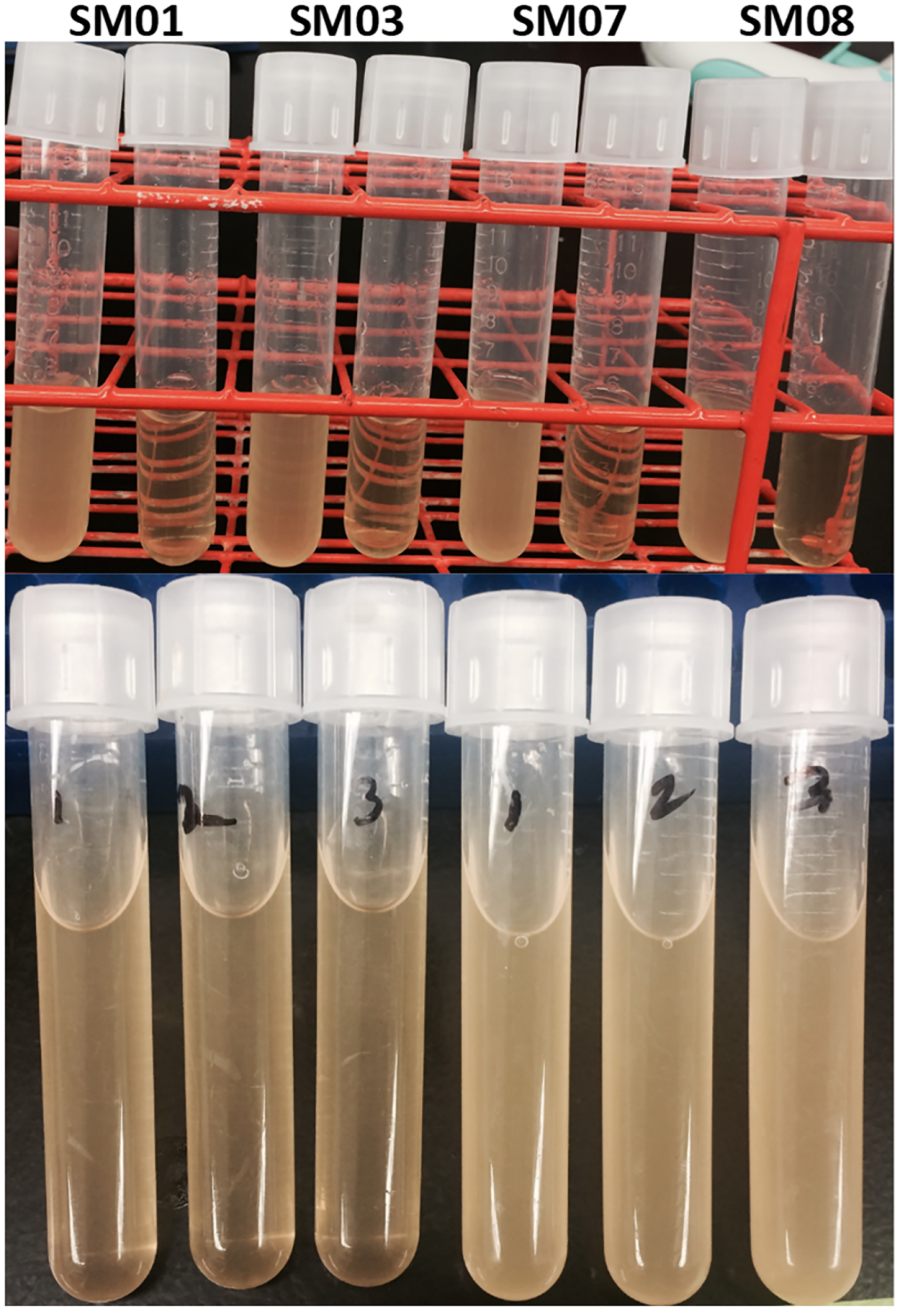
Effect of boron-based small molecules on the *Listeria monocytogenes* LapB mutant. Addition of small molecules to media prevented overnight growth of the *Listeria* mutant (upper panel). Addition of small molecules to overnight cultures of the *Listeria* mutant (first three cultures) resulted in bacterial lysis (lower panel).

Small molecule concentrations at 5, 10, and 25 μM had no significant effect on the growth of *L*. *monocytogenes* wild-type. However, 50 μM concentration resulted in slight inhibition of growth (Fig 3A). When bacterial viability was checked, colony numbers correlated with the OD readings, except that 25 μM caused slight viability decrease (Fig 3B). Therefore, all muscle attachment experiments were conducted at 10 μM concentration. Because *Listeria* LapB mutant exhibited decreased or no growth and lysis in presence of small molecules (Fig 4), it was not included in the fillet attachment experiments.

### Effect of boron-based small molecules on *Listeria monocytogenes* muscle attachment

**Fig 5:**
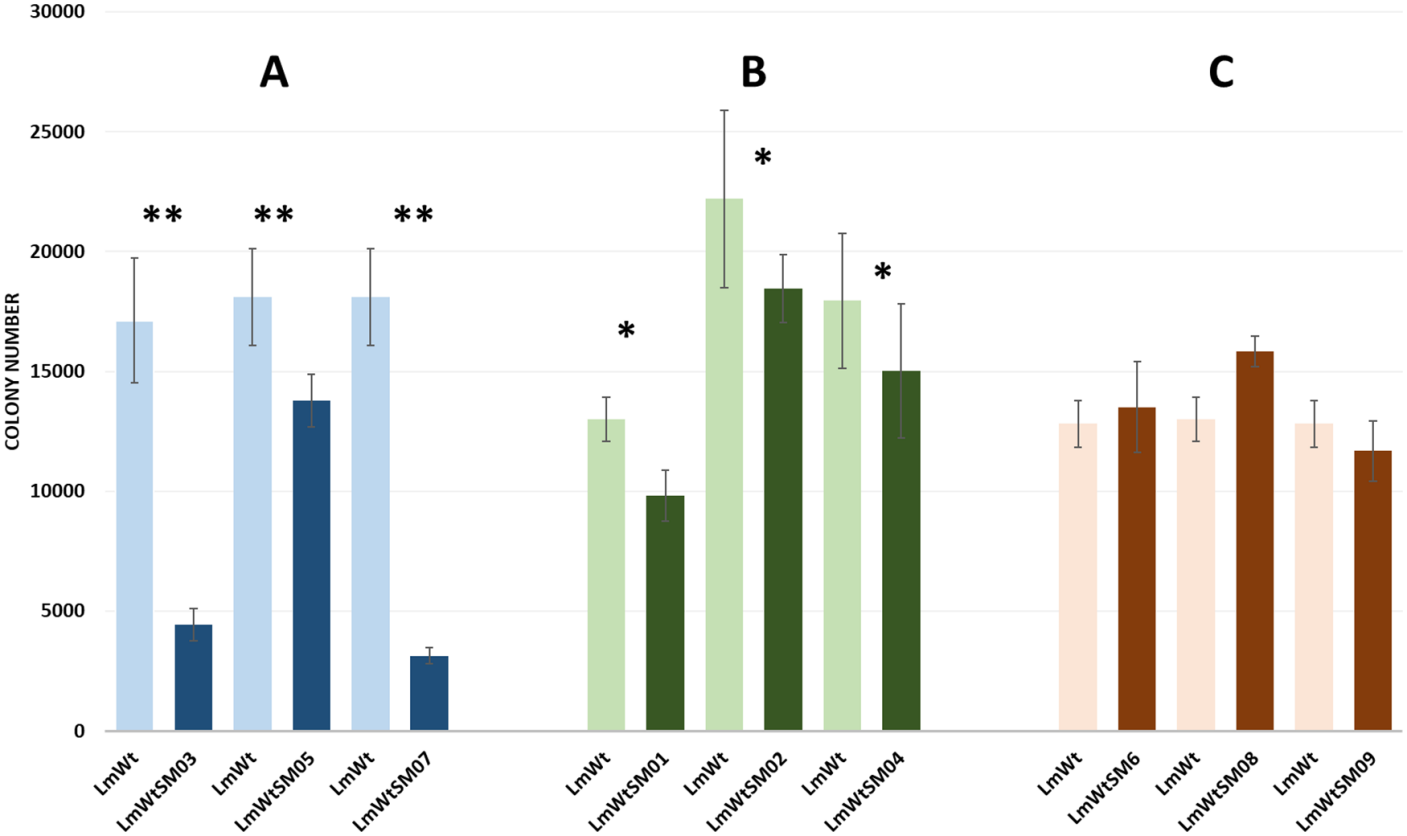
Effect of boron-based small molecules on *Listeria monocytogenes* attachment to catfish muscle tissue. Highly effective (**) boron-based small molecules (P < 0.001) (A), effective (*) boron-based small molecules (P values < 0.005) (B), and not effective boron-based molecules (P > 0.05) (C).

The boron-based small molecules significantly affected attachment of *L*. *monocytogenes* on catfish fillets. SM3, SM5, and SM7 were highly effective in preventing *L*. *monocytogenes* attachment to catfish muscle (Fig 5A). Also, SM1, SM2, and SM4 significantly reduced *L*. *monocytogenes* attachment (Fig 5B). On the other hand, SM6, SM8, and SM9 showed no significant decrease in *L*. *monocytogenes* attachment compared to control (Fig 5C).

## Discussion

LapB protein is involved in *L*. *monocytogenes* pathogenesis and potentially mediates attachment to host cell surfaces. The purpose of the current research was to design boron-containing small molecules targeting the LapB protein and evaluate their effect on *L*. *monocytogenes* attachment to catfish muscles. A total of nine boron-based small molecules were synthesized, six of which caused significantly reduced muscle attachment of *L*. *monocytogenes*.

To target LapB protein, nine different compounds were synthesized with different pharmacophore groups. Two molecules (SM3 and SM7) showed the highest reduction in the attachment. SM1, SM2, and SM5 have attached boron groups, and only SM5 has a significant effect on reduction of attachment. SM4 and SM8 both have a Potassium-Fluorine-Boron (K-F-B) group, and they have small or no effect on *L*. *monocytogenes* attachment. Finally, SM6 and SM9, which are much smaller molecules than the other boron-based compounds, show the lowest level of reduction in *L*. *monocytogenes* attachment. Thus, Boron-Oxygen-Boron (B-O-B) and Boron-Hydroxyl-Boron (B-OH-B) structured molecules might bind to the active site of LapB when they have a Nitric oxide (NO) ring structures.

Development of boron atom containing new pharmacological agents is a frontier area in drug discovery. Proof of this concept is provided by FDA approved boron containing compounds for cancer (Velcade) and antifungal therapy (Tavaborole and Crisaborole). Another significance to using boron-based pharmacological agents in drug discovery is due to reactive oxygen species (ROS) scavenger property of boron atom. Based on this concept several boron-based ROS activated pro-drugs are under investigation to target cancer cells and others.

LapB protein (Lmof2365_2117) is a cell wall surface anchor family protein in *L*. *monocytogenes* F2365 [34]. It is located between phosphotransferase enzyme family protein and a putative DNA-binding protein in the chromosome. It was previously determined that adherence of F2365Δ*2117* to Caco-2 cells was significantly lower compared to wild-type [24]. The mutant strain’s replication in intestinal epithelial cells and murine macrophages was also impaired [24]. These results suggest that boron-based molecules may have an effective binding capacity to *Listeria* LapB protein, which blocks the attachment of *Listeria* to catfish muscle.

In another study, the expression of the surface protein LapB was studied in different serotypes of *Listeria spp* to develop Anti-LapB Monoclonal Antibodies for identification and detection of virulence strains [35]. Also, the regulation of virulence genes is studied in *L*. *monocytogenes* by focusing on sRNAs between 50–300 bp [36]. A group of small RNAs named as LhrC has a role in the regulation of LapB protein in *L*. *monocytogenes* [37]. LhrC controls the level of OppA and other virulence-associated cell envelope proteins in *L*. *monocytogenes* via four additional copies of LhrC [38]. In *L*. *monocytogenes* F2365, there is only one copy of LhrC sRNA. Thus, further studies are needed to explain the transcriptional and post-transcriptional regulation of LapB.

Interestingly, *Listeria* LapB mutant exhibited reduced or no growth and lysis in presence of small molecules. Currently, underlying mechanisms of these observations are unknown. It is possible that absence of LapB might have effect in *Listeria* membrane integrity, which may lead to increased intake of small molecules. Excess small molecules might target other bacterial molecules affecting bacterial metabolism, membrane integrity or bacterial growth. RNA chaperones that facilitate sRNA-mRNA interactions might interact with small molecules and this will have global effect on bacterial gene expression. Further, LhrC mentioned above is a small RNA that controls expression of LapB and other membrane-associated proteins, In the mutant strain, small molecules may have better access to LhrC, affecting bacterial cell integrity.

This work suggests that boron-based small molecules designed to interact with the potential active site of *Listeria* LapB seem to be efficient in reducing the attachment of *L*. *monocytogenes* to catfish muscle, which could provide an alternative strategy for reducing *L*. *monocytogenes* contamination in fresh and frozen food products.
